# Heat Source Modeling and Residual Stress Analysis for Metal Directed Energy Deposition Additive Manufacturing

**DOI:** 10.3390/ma15072545

**Published:** 2022-03-30

**Authors:** Abhilash Kiran, Ying Li, Josef Hodek, Michal Brázda, Miroslav Urbánek, Jan Džugan

**Affiliations:** COMTES FHT a.s., Průmyslová 995, 334 41 Dobřany, Czech Republic; ying.li@comtesfht.cz (Y.L.); josef.hodek@comtesfht.cz (J.H.); michal.brazda@comtesfht.cz (M.B.); miroslav.urbanek@comtesfht.cz (M.U.); jan.dzugan@comtesfht.cz (J.D.)

**Keywords:** additive manufacturing, directed energy deposition, moving heat source, thermal stress, goldak model, concentrated heat source, residual stress

## Abstract

The advancement in additive manufacturing encourages the development of simplified tools for deep and swift research of the technology. Several approaches were developed to reduce the complexity of multi-track modeling for additive manufacturing. In the present work, a simple heat source model called concentrated heat source was evaluated for single- and multi-track deposition for directed energy deposition. The concentrated heat source model was compared with the widely accepted Goldak heat source model. The concentrated heat source does not require melt pool dimension measurement for thermal model simulation. Thus, it reduces the considerable time for preprocessing. The shape of the melt pool and temperature contour around the heat source was analyzed for single-track deposition. A good agreement was noticed for the concentrated heat source model melt pool, with an experimentally determined melt pool, using an optical microscope. Two heat source models were applied to multi-track 3D solid structure thermo-mechanical simulation. The results of the two models, for thermal history and residual stress, were compared with experimentally determined data. A good agreement was found for both models. The concentrated heat source model reported less than the half the computational time required for the Goldak model. The validated model, for 3D solid structure thermo-mechanical simulation, was used to analyze thermal stress evolution during the deposition process. The material deposition on the base plate at room temperature results in lower peak temperatures in the layers near the base plate. Consequently, the higher thermal stress in the layers near the base plate was found, compared to the upper layers during the deposition process.

## 1. Introduction

Additive manufacturing (AM) is growing rapidly for the application of large-scale production. AM is in the center focus, due to its limitless design manufacturability, various customized material deposition, and ability to automate the manufacturing process, which are few key factors that capitalize over traditional manufacturing methods. AM has achieved immense growth, due to progress in several fields, such as numerical simulations, materials modeling, software development related to AM, customized powder materials preparation for AM, characterizing techniques, and so on [1,2,3,4,5]. Virtual engineering, with the help of numerical modeling, is a powerful tool for the swift progress of AM technology.

The performance of AM components depends on several process parameters, such as heat source power, deposition rate, thermal history, etc. The advantage of the finite element method (FEM) process simulation is that each process parameter can be varied, and its impact or role on the quality of deposition can be evaluated. Thus, it helps to understand the deposition process and optimize the process parameters. The numerical simulation serves to minimize the failure rate of the built structure, improves the quality of the structure, and helps to understand the complex phenomena that govern the AM to success. FEM simulation serves as a strong tool to identify and understand the critical phenomenon, which cannot be observed or visualized during the real-time deposition in micro, meso, or macro scales.

The concentric high-energy heat source is utilized for material deposition during metal AM processes like selective laser melting (SLM) and directed energy deposition (DED). The high-energy concentric heat source creates a large temperature gradient around the melt pool. Therefore, residual stress and thermal distortion in the built structure are inevitable for AM parts [6,7]. Residual stresses are commonly associated with unexpected failures, affecting the strength of the built structure, fatigue life, and dimensional inaccuracy [8,9]. This shows that the thermal history and temperature distribution, during deposition, are major factors that influence the quality of the deposited structure.

Thermal history prediction for thermo-mechanical simulation plays a vital role in the calculation of residual stresses and structures distortion [3,10]. Precise thermal history calculation is the first key step for the successful implementation of a numerical model in AM process simulation. The heat source definition is crucial function for thermal simulation. The thermal heat source defines the temperature distribution in and around the melt pool. The volumetric heat source model is widely applied for welding and AM. The research on the heat source model dates back to the 1940s. A mathematical model of moving heat source was proposed for 3D analysis [11]. The double ellipsoid distribution model developed by Goldak et al. [12] is widely accepted for welding simulation, such as shielded metal arc welding (SMAW), flux-cored arc welding (FCAW), submerged arc welding (SAW), plasma arc welding (PAW), and gas metal arc welding (GMAW) [13,14,15,16]. Numerous application in building thermomechanical simulation for distortion evaluation for Ti-6Al-4V in directed energy deposition [17]. Goldak model applications were reported for evaluating residual stress in the build structure for AM [18,19].

The precise definition of heat source is an important factor to determine the fusion zone (FZ), heat-affected zone (HAZ), temperature distribution around the deposition track, and peak temperature [20,21]. This model requires several important melt pool parameters to define heat flux magnitude and distribution accurately.

The wide applicability of the Goldak model is due to the customized definition of the double ellipsoid heat source distribution. The power densities, energy input rate, fractional factors for front and rear quadrant and several parameters (a, b, cf, cr, ff, fr) in the model, helps to define heat source, effectively for the respective methods. Double ellipsoid heat source parameters need to be determined from the weld pool created from the respective technique [22,23]. The weld pool dimensions varies, due to changes in process parameters and different operating conditions [24,25,26,27]. The experimental determination of heat source parameters, due to change in process parameters and/or for different techniques is time-consuming and expensive. Researchers reported relations between the Goldak’s parameters to reduce the complexity by reducing the double ellipsoid heat source parameters [15,22]. Similarly, research on reducing the complexity of the heat source model was reported [10,28].

Welding contains a limited number of deposition tracks, as compared to AM, whereas AM contains several thousand weld tracks that were overlapped. Instead of transient a concentric heat source, researchers introduced the heat flux to an entire layer in a step. The heat was introduced layer after layer called the “thermal cycle” heat input method [10]. The accuracy of the residual stress estimation was preserved for heat input for multiple layers in one step. This method is called the lumping layer. The thermal cycle heat input and lumping layers method were successful in predicting residual stress in the deposited structure. These methods were efficient in computational cost reduction; whereas, it ignores the localized heat and cooling phenomenon.

Similarly, Reddy et.al suggested a simple heat source model for wire arc additive manufacturing (WAAM) [29]. They interpreted that the Goldak heat source model does not influence the thermal and mechanical results and high computational cost for the renowned model. Therefore, the author suggested for simple uniform heat distribution model importance on multi-layered WAAM.

In the present work, a simple moving heat source is applied to the single- and multi-track simulation. The goal is to evaluate its efficiency for AM. The simple volumetric heat source distribution is called the concentrated heat (CH) source. According to the author’s knowledge, the CH source model for DED metal AM was not reported. This method does not require numerous parameters to define in the thermal simulation. The intensity of the heat source is governed by the defined heat source power and absorption efficiency. Similar to the “thermal cycle” heat input method, which approximately applied heat flux in a layer, the CH model approximates the heat flux distribution density within the deposition track.

The accuracy of the CH source model was compared with the Goldak double ellipsoid heat source model. The numerical results were compared to the experimentally measured thermal and residual stresses, respectively. The thermal distribution in the deposit material, from both models, was compared during single-track deposition. In the next stage, these two thermal models were applied to the 3D structure simulation for residual stress calculation. The CH source model accuracy was compared with the Goldak model. Both numerical results were validated with experimentally determined residual stress using the contour method.

## 2. FEM Model

The thermomechanical FE model for DED was constructed and post-processing was done using the 3DExperience and Abaqus version 2019 software (Dassault Systèmes, Vélizy-Villacoublay, France), respectively. The residual stress generated during material deposition was calculated in two steps. The first step in the uncoupled thermo-mechanical simulation is the transient thermal analysis, which was performed to calculate time-dependent temperature distribution. Secondly, the temperature field output from the thermal analysis at each time step is employed to calculate the stress field in subsequent structural analysis. The numerical simulation was conducted with the use of the computer powered by Intel i7 six-core processor (3.20 GHz) with 32 GB random access memory (RAM).

### 2.1. Thermal Analysis

Transient heat input method was employed to define the melt pool generated by the laser heat source. The elements were activated according to the time and coordinates in the activation series, using the element activation keyword. The activated elements at a particular location in the model contribute to the analysis. The inactive elements are not considered for calculations. The governing equation for transient heat transfer analysis is given by

(1) $$\rho c\frac{\partial T}{\partial t}\left(x,y,z,t\right)=-\nabla .\;\vec{q\;}\left(x,y,z,t\right)+Q\left(x,y,z,t\right)$$

where ρ is the density, c specific heat capacity, T is the temperature, $\left(x,y,z\right)$ are coordinates, t is the time, ∇  is special gradient, $\vec{q\;}$ is the heat flux, and Q is the heat generation.

### 2.2. Mechanical Analysis

The temperature distribution from the thermal analysis is the driving factor for mechanical analysis. Mechanical response is calculated for residual stress estimation during deposition process and at the end state after structure cool down to room temperature. Body force, due to gravity, is ignored in the present calculation. The relationship between stress and strain is defined as [30]:

(2) $$\left\{\sigma_{ij}\right\}=\left[D\right]\left\{\varepsilon^{e}\right\}$$

where $\sigma_{ij}$ is the stress vector, D is the elasticity matrix, and $\varepsilon^{e}$ is the elastic strain vector.

The strain induced in the structure is defined as follows [10]:

(3) $$\varepsilon^{total}=\varepsilon^{e}+\varepsilon^{p}+\varepsilon^{t}$$

where $\varepsilon^{total}$ is the global deformation during mechanical analysis, $\varepsilon^{e}$ is the elastic strain, $\varepsilon^{p}$ is the plastic stain, and $\varepsilon^{t}$ is the thermal strain.

The thermal strain is computed as [31]:

(4) $$\varepsilon_{t}=\alpha \left(T-T_{ref}\right)$$

where α is the thermal expansion coefficient, and $T_{ref}\;$ is the reference temperature, at which thermal strain is assumed to be zero.

The plasticity during mechanical calculation was considered. The relation is defined as [19]:

(5) $$f\left(\sigma_{ij}\right)=f_{0}\left(\varepsilon^{p},T\right)$$

where $f\left(\sigma_{ij}\right)$ is the yield function, $f_{0}$ is the yield stress function depend on temperature (T) and plastic strain ($\varepsilon^{p}$).

### 2.3. Boundary Condition

Progressive cooling, due to convection and radiation, was applied to the base plate and depositing elements. The previous elements were covered over the time when the new elements are added. The activated new element surface, which is exposed to the ambient environment, was continuously tracked at the start of an increment. The convection and radiation conditions were applied to the evolving free surfaces at any given time. Similarly, the heat loss condition was not applied to the unexposed element faces. The convection and radiation heat loss were derived from the literature [8]. The heat transfer coefficient (HTC) of 18 W/m^2^·K and emissivity of 0.1 were applied during thermal simulation. The material was deposited with laser power of 500 W for both single- and multi-track 3D solid structure. Therefore, the laser power of 500 W was set for thermal simulation. The initial temperature of 27 °C was set as the ambient condition.

Evolving free surface heat transfer boundary conditions, such as convection and radiation, were applied on the exposed elements facet area. Heat flux on a surface, due to convection, is governed by:

(6) $$q_{c}=-h_{c}\;\left(T-T_{s}\right)$$

where $q_{c}$ is the heat flux across the surface, $h_{c}$ is the reference film coefficient, T is the temperature on the surface, $T_{s}$ is the reference sink temperature. Further, $h_{c}$ is described by

(7) $$h_{c}=\frac{N_{u}K_{f}}{L}$$

where $N_{u}$ is the dimensionless Nusselt number, $K_{f}$ is the thermal conductivity of the fluid, and L is the characteristic length.

Heat flux on the surface, due to radiation to the environment, is governed by [32]:

(8) $$q_{r}=\sigma \epsilon \left(T^{4}-T_{0}^{4}\right)$$

where $q_{r}$ is the heat flux across the surface, ϵ is the emissivity of the surface, σ is the Stefan-Boltzmann constant, $T_{0}$ the ambient temperature, and T the temperature at this point of consideration.

### 2.4. Heat Source Modeling

The two thermal heat source models are explained in this section.

#### 2.4.1. Goldak Model

**Figure 1:**
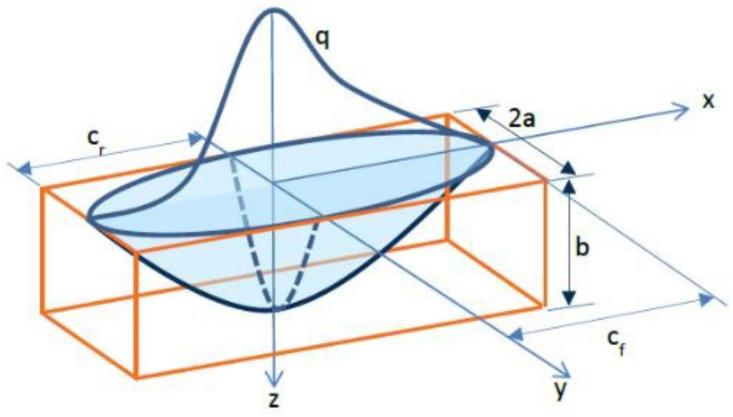
Representation of Goldak double ellipsoidal heat source (Dassault Systèmes, Vélizy-Villacoublay, France).

Heat flux distribution in the melt pool was simulated using a Goldak heat source model. The heat source model defines the heat generated per unit volume in the molten pool region. The double ellipsoidal heat source model is shown in Figure 1. It is widely used for the thermal simulation of the moving heat source in AM [19]. The movement of the heat source during material deposition generates inconsistent energy distribution. This model takes the uneven heat energy distribution into account. The heat energy was distributed, as per the model definition to element nodes, in the form of heat flux density.

Power density distribution is given by the below equation:

(9) $$q_{f/r}=\frac{6\sqrt{3}\;f_{f/r}}{abc\pi \sqrt{\pi }}\;Q\;e^{\left(-\right.\frac{3x^{3}}{c_{f/r}^{2}})}\;e^{\left(-\right.\frac{3y^{2}}{a^{2}})}\;e^{\left(-\frac{3z^{3}}{b^{2}}\right)}$$

where *a*, *b*, *c_f_*, *c_r_* defines the variables that define axes of the ellipsoidal heat source. Q is the heat source power. $f_{f/r}$ defines the energy flow intensity. Subscript “*f*” represents the region front portion of the melt pool and “*r*” represents the region rear region of the melt pool from the origin of the axes as depicted in Figure 1. It is fulfilled by the following condition:

(10) $$f_{f}+f_{r}=2$$

#### 2.4.2. CH Source Model

The intensity of the heat flux distribution is not defined in a specific direction at the given time in the CH model. CH source model approximates the laser spot as a concentrated moving heat flux. It is a simple heat source distribution algorithm to specify the point energy source along the defined laser path. The intensity of the heat flux was controlled by the power of the laser source. In the present work, laser power was set to 500 W, taken from the experiment. The heat flux is applied to the elements in the focus of the moving heat source. The heat flux is distributed around the defined path. The path of the heat source was defined by time and special coordinates. The special coordinate creates the moving paths within the meshed CAD model volume. These paths within the CAD model intersect with the elements. The heat flux is applied to the elements, which intersects from the defined scanning path.

### 2.5. Meshed Model

**Figure 2:**
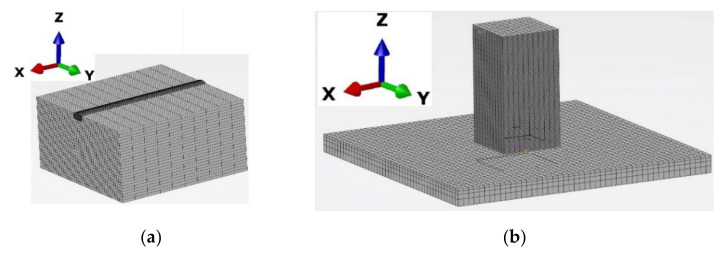
FEM meshed model: (**a**) Single-track; (**b**) Solid 3D structure.

A 3D solid DC3D6 and DC3D8 elements from the 3D Experience library were used to create a fine mesh for single-track simulation. A fine mesh was generated in and around the deposition area for a single-track deposition to capture a steep temperature gradient at a specific time step. The FE model consists of 75,520 elements and 85,382 nodes depicted in Figure 2a.

Linear heat transfer elements (DC3D8) were selected for the thermal simulation. Linear elements (C3D8) from the 3DExperience library were used for mechanical analysis. The FE model for the 3D solid structure consists of 28,576 elements (2 mm mesh size) and 35,189 nodes depicted in Figure 2b.

Progressive material deposition with respect to time and corresponding position was considered during both a thermal and a mechanical simulation. The progressive material deposition was implemented using progressive element activation. The elements were activated in each successful increment. The elements were activated by assigning a volume fraction. The volume fraction was assigned to an element at the beginning of each increment. The status of an element can be changed from inactive to fully active (i.e., material volume fraction equal to 0 or 1). The elements in the deposition structure were activated progressively. During the analysis, any element that is defined as activated is filled with material, whereas the elements which are not considered for calculation were considered inactive. For example, during deposition of first layer elements representing remaining upper layers assigned as empty (inactive) as per the timeline.

The time and special coordinates of the transient heat source movement were defined using an external data file along with the laser power at that specific time period. The interlayer dwell time and idle movement of the laser were considered during the simulation. The idle laser head movement between the two points was defined by laser power. The laser status was turned on by assigning corresponding laser power from the experiment data. During the interlayer dwell time and idle movement of the laser, the laser power was set to zero. This set of data was generated from the G-code used for the DED machine. Similarly, the material deposition sequence is defined in an attached file (in the form of time and spatial coordinates corresponding to a laser source movement) to add material for the specific element in a given time increment.

## 3. Materials and Methods

**Figure 3:**
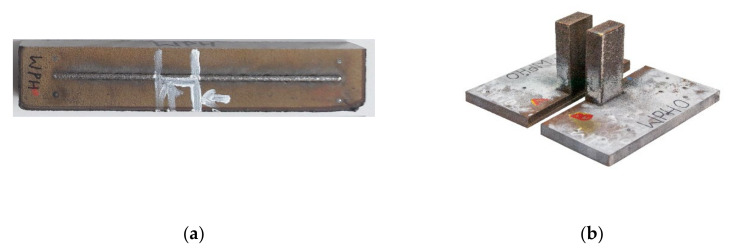
(**a**) Single-track deposition (**b**) Multi-track cuboid structure.

The InssTek MX-600 metallic deposition system (InssTek, Daejeon, South Korea) with a 2 kW Ytterbium fiber laser was used for the material deposition [10]. The machine is equipped with DED technique. The two heat source models were evaluated on single- and multi-track austenitic stainless steel 316 L material, deposited using DED technique. The aim of implementing heat source models for single-track simulation is to examine the melt pool generated from the respective cases. It is convenient and possible to precisely evaluate the melt pool with the experimental data for the single-track. The temperature contour around the moving melt pool and the shape of the melt pool generated from the models were analyzed. The top view of the single-track deposition and base plate is shown in Figure 3a.

The ultimate goal of this work is to evaluate the accuracy of the numerical models and CH model implementation for AM thermo-mechanical simulation. Therefore, a 3D solid structure with 160 layers was deposited. The cut solid structure for thermal stress evaluation is depicted in Figure 3b. It has a cross section are 20 × 20 mm^2^ and a height of 40 mm. The process parameters are listed in the Table 1. The process parameters were derived from the literature [33].

Austenitic stainless steel 316 L was selected as the material for the powder and base plate. Austenitic stainless steel 316 L does not undergo phase transformation in the solid state. The carbon content in the deposition powder is 0.016 (wt.%). The chemical compositions of the powder used for deposition and base plate material are listed in Table 2.

**Figure 4:**
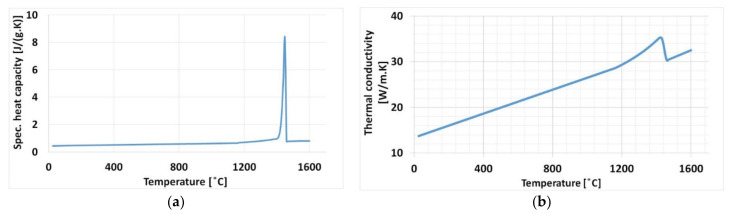
Temperature-dependent material properties (**a**) Specific heat capacity (**b**) Thermal conductivity [10].

Temperature dependent material properties were considered for thermo-mechanical simulation. For the present work coefficient of thermal expansion and young’s modulus were obtained experimentally. Both measurements are described in detail in the results sections. The other properties, such as thermal conductivity, specific heat capacity, latent heat, and plasticity data, were derived from the literature [10]. The specific heat capacity and thermal conductivity from the literature are shown in Figure 4. The temperature dependent material properties were applied to both deposited material and base plate.

### 3.1. In Situ Temperature Measurement

The experimental method for the temperature recording during deposition and residual stress calculation in the deposited 3D solid structure is explained in the following sections.

The temperature on the base plate was measured using the thermocouple type “K”. The temperature was recorded at equal intervals during deposition and cooling time periods [8].

**Figure 5:**
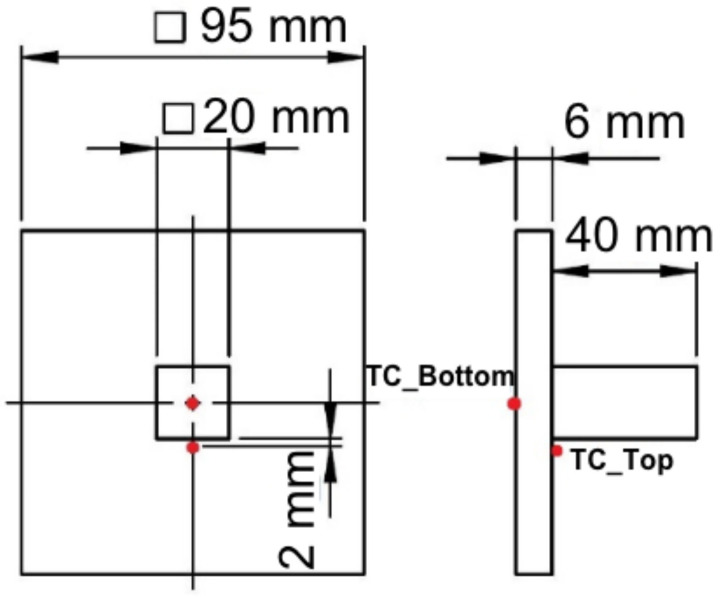
Structural dimensions for 3D solid structure and thermocouple locations.

A solid structure with dimension 20 × 20 × 40 mm^3^ was deposited on the base plate of dimension 95 × 95 × 6 mm^3^. Two thermocouples were welded on top (TC_Top) and bottom (TC_Bottom) of the base plate. The thermocouple welded on the top surface of the base plate was placed around 2 mm away from the contour of the deposit structure. The bottom thermocouple was welded at the center of the base plate. The position of the thermocouples is marked in the red dot shown in Figure 5, whereas the numerical results for the single-track were validated using melt pool dimension captured using an optical microscope.

### 3.2. Contour Method

The destructive contour method was used to estimate the residual stress inside the deposited structure [34,35]. The deposited part with the base plate was cut at the center using a wire electric discharge machine (diameter of the wire 0.25 mm). The cut structure considered for residual stress calculation is depicted in Figure 3b.

The residual stress perpendicular to the cut plane was calculated to compare with the numerical simulation results. The detailed description of contour method comparison with AM can be found in the literature [8].

## 4. Results and Discussion

The detailed investigation on the temperature dependent material properties for numerical simulations are presented in this section.

The crucial Goldak parameters were determined for thermal model. The relevant resources were presented in this section. The thermal simulation for single-track results were compared with the microscopic images. The efficiency of the melt pool size determined from the two-heat source numerical models were reported in this section. The thermal stress calculated for 3D solid structure from numerical models were compared with experimentally determined using contour method. The internal stress evolution over the deposition time was analyzed for specific layers. Nodal stress from the numerical model output was extracted at the specific layers and correlation with the thermal filed at the specific node was reported.

### 4.1. Coefficient of Thermal Expansion (CTE)

**Figure 6:**
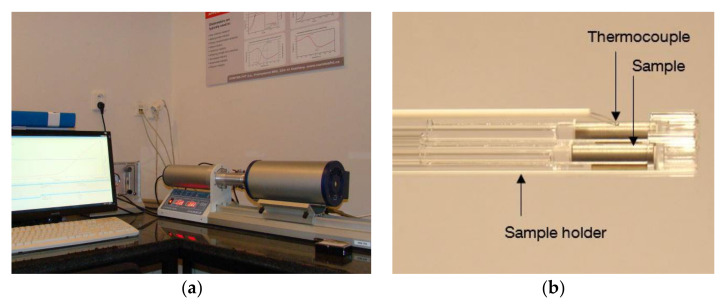
(**a**) Dilatometer set up. (**b**) Sample holding system.

The thermal expansion coefficient was measured using dilatometer L75 PT (linseis messgeraete GmbH, Vielitzerstrsse, Germany). The sample for the thermal expansion coefficient was taken from the additively manufactured structure. Samples were cut in the cylindrical shape of diameter 5 mm and length 10 mm. One sample was cut in the horizontal plane in *x*-axis and another one is in *z*-axis direction to take into account directional properties (where *z*-axis is the build direction). The heating and cooling rate were set to 10 °C/min and the maximum temperature was 1300 °C. The dilatometer, with measuring set up, is shown in Figure 6a. Samples were placed horizontally with a contact force of 0.3 N, as shown in the picture Figure 6b.

Relative elongation was measured over the temperature rise and drop. Relative elongation in % depending on temperature was evaluated from the equation,

(11) $$\varepsilon \left(T\right)=\frac{\Delta l}{l_{25}}$$

where, Δl is length change during heating between 25 °C and given temperature, l_25_ is initial sample length at 25 °C.

Linear thermal expansion coefficient at the given temperature is calculated from the following equation,

(12) $$\alpha \left(T\right)=\frac{1}{l_{25}}\frac{dl}{dT}$$

where, $\frac{dl}{dT}$ is derivative of the length change at a given temperature.

**Figure 7:**
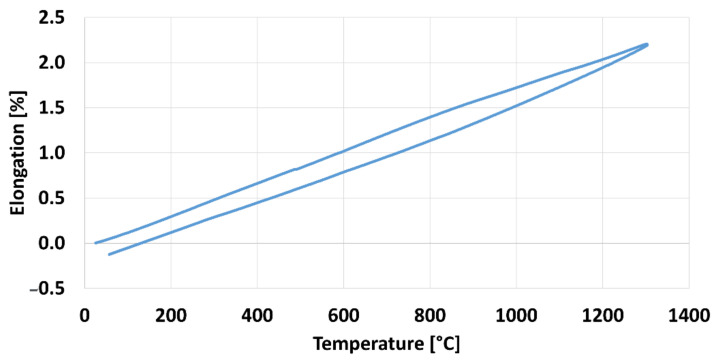
Elongation of sample during heating and cooling phase.

Elongation went approximately the same way during heating and cooling due to no phase transformation for austenitic stainless steel 316 L material sample. The percentage of elongation during heating and cooling from room temperature to 1300 °C is depicted in Figure 7. The upper slope was during heating phase and lower slope was during cooling of the sample. The slope for both phase remains same and no sudden shift due to phase transformation was recorded.

The 3DExperience software requires total or average thermal expansion. Average elongation was calculated, and the regression polynomials were fitted for the two samples data. Strain from regression polynomials for the corresponding temperature were calculated. Total or average thermal expansion was calculated using the following equations for all corresponding temperatures.

(13) $$\alpha_{1}=\varepsilon_{1}^{th}/\left(T_{1}-T_{0}\right)$$

(14) $$\alpha_{2}=\varepsilon_{2}^{th}/\left(T_{2}-T_{0}\right)$$

(15) $$\alpha_{3}=\varepsilon_{3}^{th}/\left(T_{3}-T_{0}\right)$$

**Figure 8:**
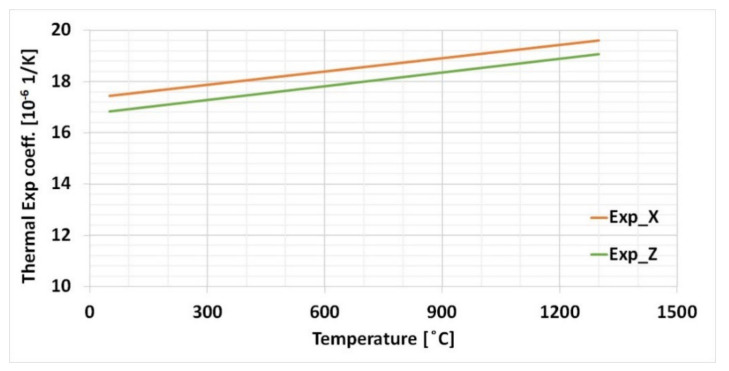
Temperature dependent thermal expansion coefficient plot.

The average thermal expansion, calculated from the above equations, for two sets of samples orientation is show in Figure 8.

The CTE for two sets of samples calculation, depicted in Figure 8, show a similar slope for samples orientated in the *x*- and *z*-axis. Therefore, one set of the plot values were considered for numerical simulation.

### 4.2. Young’s Modulus

**Figure 9:**
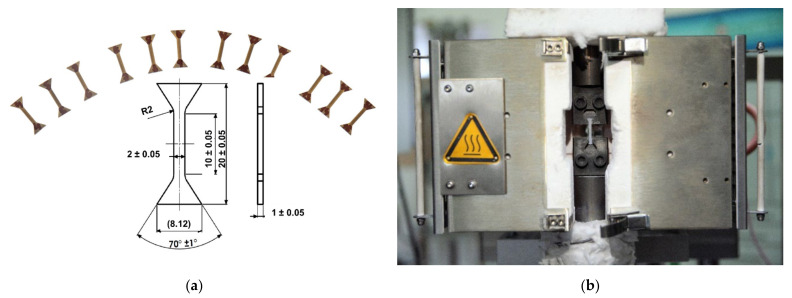
(**a**) Miniaturized tensile test specimens and corresponding geometry dimensions (**b**) Testing setup.

The tensile properties were investigated by miniaturized tensile test (MTT) [36,37]. The testing specimens were extracted from the additively manufactured part by a wire electric discharge machine (WEDM), in accordance with the geometrical dimensions depicted in Figure 9a. After extraction, the specimens were polished, in order to reach the required surface roughness. The heating furnace and sample holding system is show in Figure 9b.

The miniaturized tensile tests were performed using a universal testing machine with a linear drive ZWICK/ROELL-Z250 equipped with a load cell with a capacity of 2.5 kN. The tests were conducted under quasi-static conditions (a strain rate of 0.00007 s^−1^) at room and elevated temperatures (200, 400, 600, and 800 °C). The testing procedure followed the standards ISO 6892 (metallic materials—tensile testing—Part 1: method of test at room temperature; Part 2: method of test at elevated temperature). The strain was measured by using a digital image correlation (DIC) method. The specimen was heated in a resistance furnace. Tests were performed after reaching the testing temperature with a hold time of ~20 min. Three samples were tested per test condition. After testing, the final cross-sectional area of each specimen was measured using a stereomicroscope. Based on all the measured values, the characteristic tensile properties were evaluated.

**Figure 10:**
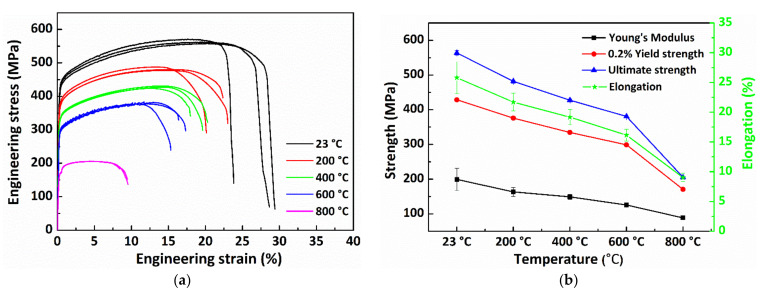
(**a**) Engineering stress-strain curves at different temperature conditions, and (**b**) Temperature dependence of Young’s modulus, yield strength, ultimate strength, and elongation for DED-SS316L xy-orientated specimens.

Engineering stress–strain curves for all xy-orientated specimens are depicted in Figure 10a. It can be seen that all test results show a good consistency. Typical ductile fracture characteristics can be observed, which are commonly seen in the tensile results for AM-parts [38,39,40,41]. In addition, temperature dependence of young’s modulus, 0.2% yield strength, ultimate strength, and elongation are also shown in Figure 10b. It is clearly indicated from the results that the values for young’s modulus, 0.2% yield strength, ultimate strength, and elongation of the sample decrease in various extent with the increase of testing temperature. Particularly, a sharp drop in all of the values is observed when the test temperature increases from 600 to 800 °C, i.e., the value for young’s modulus decreases by 17.9% (200 °C), 8.8% (400 °C), 15.8% (600 °C), and 29.4% (800 °C), compared to that of room temperature. It is worthy note that, unlike increased ductility, due to the tradeoff of strength commonly observed in the conventionally-manufactured alloy [42]. The ductility in the present study is also shown to decrease with the increase of testing temperature in the testing temperature range the utilized strain rate. Similar results can be find in the tensile studies of AM-material [43]. This indicates an AM-induced microstructure-controlled deformation mechanism, different from that of conventionally processed material.

**Figure 11:**
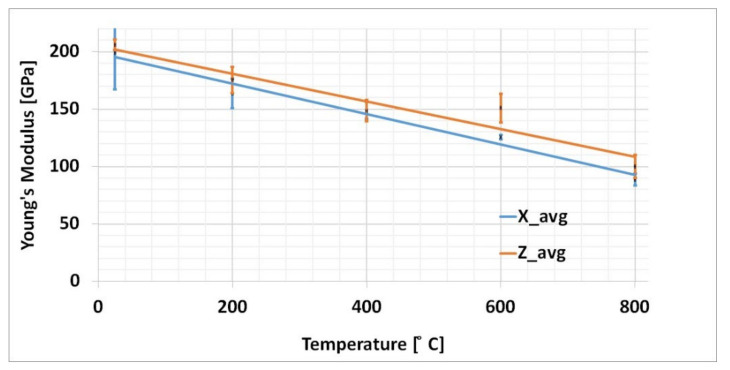
Average Young’s modulus at different temperature for samples oriented in the *X*- and *Z*-axis.

The young’s modulus for the samples orientated in two building direction are depicted in Figure 11. The young’s modulus plot for samples oriented in *x*-axis and *z*-axis has a similar slope. Therefore, one set of the plot values were considered for numerical simulation.

### 4.3. Goldak’s Parameters

**Figure 12:**
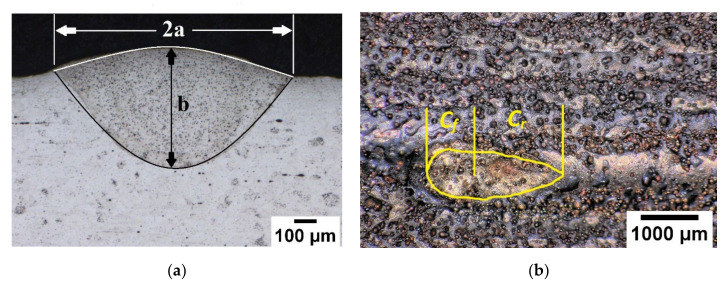
(**a**) Transverse section of the melt pool from optical microscope; (**b**) Front and rear sections based on the end crater geometry.

The Goldak parameters were calculated based on experimentally observed weld pool dimensions. The ellipsoidal heat source parameters ‘*a*’ and ‘*b*’ were measured from the transverse section of the single-track deposition, as shown in Figure 12a, whereas ‘$C_{f}$’ and ‘$C_{r}$’ were determined from the bird-eye view of the deposition depicted in Figure 12b.

The front and rear fractions of heat flux distribution were calculated using the following equation [44]

(16) $$f_{f}=\alpha \frac{C_{f}}{C_{f}+C_{r}}$$

With α = 2, $f_{r}$ was calculated using the above equation.

### 4.4. Single-Track

In this following section heat distribution of the two models is evaluated. In this section, the sizes of the molten pool predicted from two thermal model cases were compared. Both numerical results were validated with the fusion zone morphology determined from the experiment.

**Figure 13:**
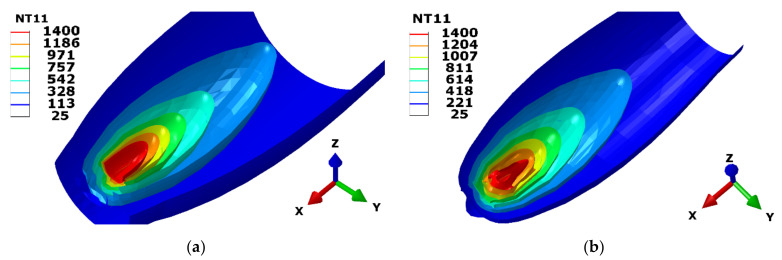
Temperature contour maps of nodal temperature in degree Celsius. Isometric view of melt pool: (**a**) Concentric heat model; (**b**) Goldak model.

The contour maps of the temperature field for both models are depicted in Figure 13. The red colored contour represents the temperature boundary of the melt pool for the 316 L steel. The melting point temperature of 1400 °C is considered as the border between melted metal in the fusion zone and non-melted base plate. The isometric view of the moving melt pool for both the model shows the temperature profile lines. The CH model generates a widespread temperature profile, whereas the Goldak model develops a narrow tail behind the melt pool.

The Goldak model creates the double ellipsoidal shape, as shown in Figure 13b, for given Goldak parameters. Interestingly, a parabolic shape appears from the front view (against to moving direction) for the CH model. A blunt curved shape was generated at the tail of the melt pool. This model develops a parabolic shape from the top view and semi ellipsoid shape in three dimensions, as shown in Figure 13a. The melt pool is symmetric about the moving axis. Understanding the melt pool shape generated by the moving heat source helps us to understand the temperature profile around the deposition area.

**Figure 14:**
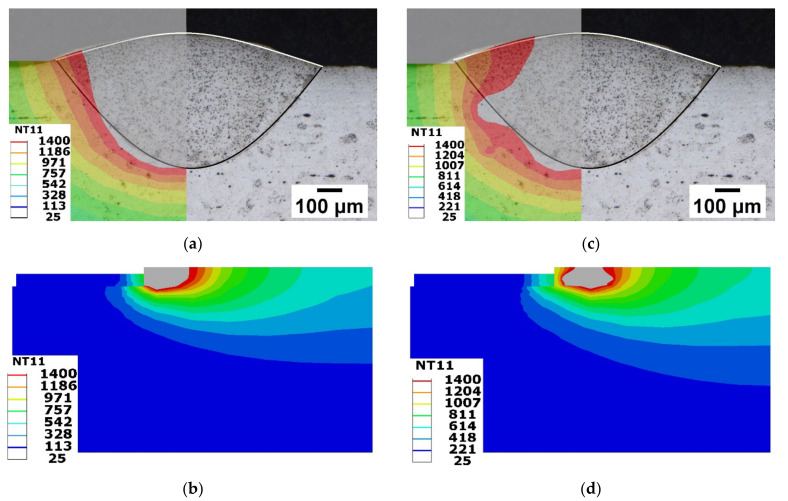
Comparison of the melt pool size between simulation and experiment: Transverse section view (**a**) Concentric heat model; (**c**) Goldak model. Longitudinal cut section view (**b**) Concentric heat model; (**d**) Goldak model.

The transverse section which is perpendicular to the moving path of single-track deposition was compared for two models, as shown in Figure 14. Figure 14a,c shows the comparison of the melt pool, obtained through simulation and experiment. The area representing temperature behind the melting point was compared with the Fusion zone. The Fusion zone, separating from the base plate, is marked with a dark line and upper surface of the deposition with a white line in the microscopic image depicted in Figure 14a,c. The CH model estimated the melt pool/Fuzion zone, with an error of 13%. CH model prediction had a slight error along the width. The error of the simulation result can be seen clearly in the transparent image depicted in Figure 14a. Similarly, the Goldak model exhibits a larger error along the width. The Goldak model estimates an error of 30%. The efficiency could be improved by optimizing the Goldak parameters. The heat source parameters depend on several factors, such as the process parameters of the respective method, base plate, and powder materials, thermal conditions, etc. It is necessary to evaluate in detail considering several factors. The peak temperature-based approach is one example that successfully predicted heat source parameters within 10% root mean square error for twin wire submerged arc welding [45]. Similarly, process specific changes need to adopt for accurate prediction of the heat source parameter to reduce error. Mohanty et al. implemented it for the alternating current square waveform arc welding with necessary changes for better parameter selection [46]. This method results in a percentage prediction error not exceeding 10%.

The present method is static analysis considering weld bead from post-processing is a simple and less time-consuming approach for approximating heat source parameters. The limitation of this static analysis is the weld pool dimension, measured in Figure 12, which is not always constant. Moving heat source material deposition is a dynamic process. Weld pool size varies over the time. This could be one of the reason for large error for Goldak method case. Considering all these factors, it shows the complexity and tedious steps involved for thermal simulation using Goldak method.

The depth of the melt pool from the simulation results was compared throughout the length of the deposition measured using optical microscope from our previous work. The cut view in the middle axis along the deposition track of the melt pool are depicted in Figure 14b,d. The melt pool generated for both the model is represented in gray color. The height of the melt pool generated by the two models was evaluated. The depth of the melt pool generated by both models throughout the length was fell within 315 μm to 510 μm. This is the value determined using the optical microscope for single-track deposition (base plate at room temperature case) [33].

### 4.5. Solid Structure

The two-heat source model was applied to a multi-layer 3D solid structure process simulation. The thermal and residual stress results were validated in this section.

#### 4.5.1. Thermal Simulation

In order to achieve the desired accuracy for the temperature field and precise residual stress calculation, temperature variation over the time at the specific node, corresponding to the thermocouple on the base plate, was compared. The thermal simulation computation times for the CH model and Goldak model were 4.10 and 8.30 h, respectively. Both model simulations were carried on the computer with the same hardware configuration. The mesh size, increment, and process parameters were maintained the same for both cases. The Goldak model took double the computational time, compared with the CH model.

**Figure 15:**
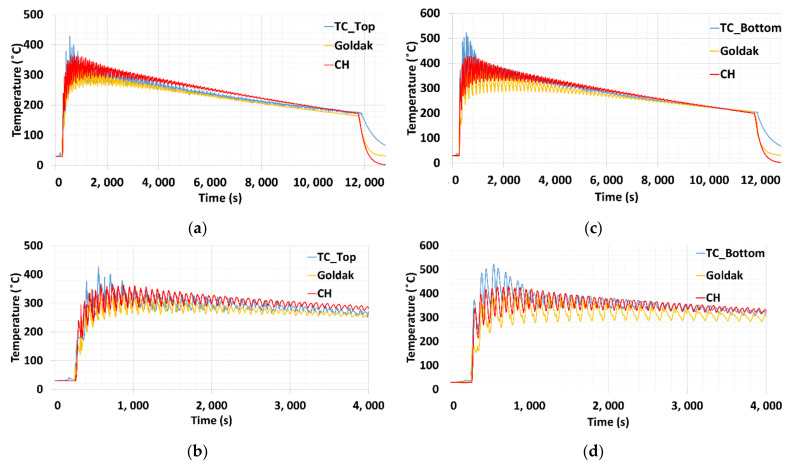
Temperature distribution on the base plate measurements (**a**) Top region (**b**) Detailed plot for the top surface (**c**) Bottom region (**d**) Detailed plot for the bottom position.

The position of the TC_Top and TC_Bottom thermocouples were depicted in Section 3.1. Thermocouples measurements at two locations were depicted in Figure 15a,c. The TC_Top recorded a peak temperature of 422 °C on the top surface of the base plate, and the temperature at the bottom reached 520 °C. This shows that the temperature developed at the bottom of the base plate is higher, whereas the temperature perpendicular to laser incidence on the surface of the base plate was always lower. Similar thermal results were reported during a cube deposition on the symmetric base plate [3]. The average temperature during the complete deposition process is higher at the bottom surface compared to the upper surface around the contour of the structure on the base plate.

As the deposition time increases the temperature on the base plate reduces. It is due to the distance of heat source interaction is increase from the base plate as the height of the deposit structure increase.

Thermal history developed from both models has a relatively close agreement with the experimentally measured data from the thermocouples, whereas a slight difference was developed between the two thermal models. The difference can be noticed in the detailed plot, depicted in Figure 15b,d, for the top and bottom positions, respectively. The difference in the peak temperature for top node is around 80 °C, and the bottom node around 100 °C. This difference could reduce with the optimized mesh size. Another possible important factor would be heat loss boundary condition. In the present case heat loss applied uniformly to entire base plate surface. Optimizing thermal boundary condition could further reduce the difference at the beginning of deposition, as well as during cooling phase, whereas the average temperature match very well during deposition process, which means that the total heat added to the system is comparable to the experiment. A 100 °C difference for welding thermal simulation was reported in an acceptable range [45]. This difference needs to be reduced for the improvement of mechanical simulation results.

#### 4.5.2. Mechanical Simulation

**Figure 16:**
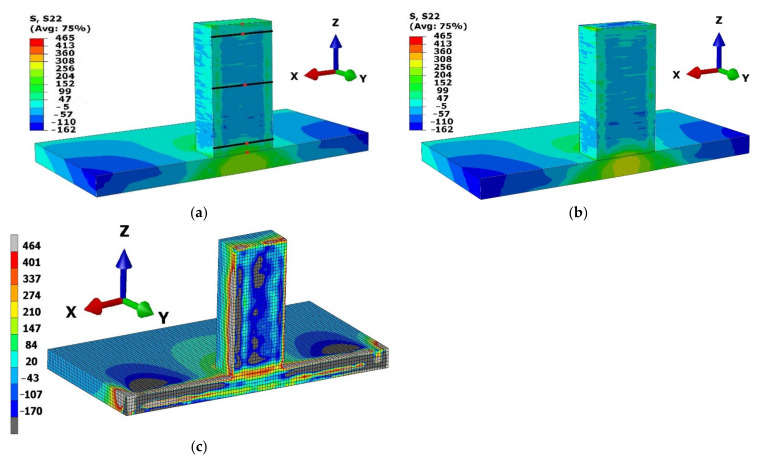
Residual stress (MPa) in Y component perpendicular to cut plane (**a**) Goldak model (**b**) CH model (**c**) Contour method.

The residual stress state in the middle section of the structure for both thermal models is depicted in Figure 16a,b. The respective thermal model’s output was considered for residual stress calculation. The mechanical model was created with the same number of mesh sizes and boundary conditions. The results achieved were independent of model preparation, process parameters, and boundary conditions. Thus, the difference in the residual stress calculation is due to the respective heat source models. The computational time required for residual stress calculation from CH and Goldak thermal output was 18 and 37.5 h, respectively.

The mechanical output from numerical calculation was sliced at the middle section, similar to the structure prepared for contour method analysis, shown in Figure 3b. The contour method results are depicted in Figure 16c. The large stress value was calculated at the edge of the deposited structure. It is certain in the contour method as the edges of the structure were influenced during cutting using wire electric discharge machine [10].

The stress components perpendicular (Y-component) to the cut surface was calculated in the contour method. A large distortion in the base plate towards Z-component was developed in the base plate around the deposition area. A similar result of base plate distortion was reported for symmetric base plate [3]. The higher stress value in the base plate was due to large Z-component distortion consider for calculation of Y-component stress. This article is focused on the residual stress in the deposited structure. Therefore, for the precise comparison with the numerical results, three paths were considered in the deposited structure. The three paths were depicted with a dark line in Figure 16a. The first path is near to the base plate (Path-1), the second is at the middle of the deposited structure (Path-2) and the third is near to the top surface (Path-3). The first path is located at a height of 3 mm from the base plate surface. This path corresponds to the 12th layer deposition. The middle path is at a height of 18.75 mm where the 75th layer was deposited. The topmost path is located at 35.5 mm correspond to the 142nd layer deposition.

**Figure 17:**
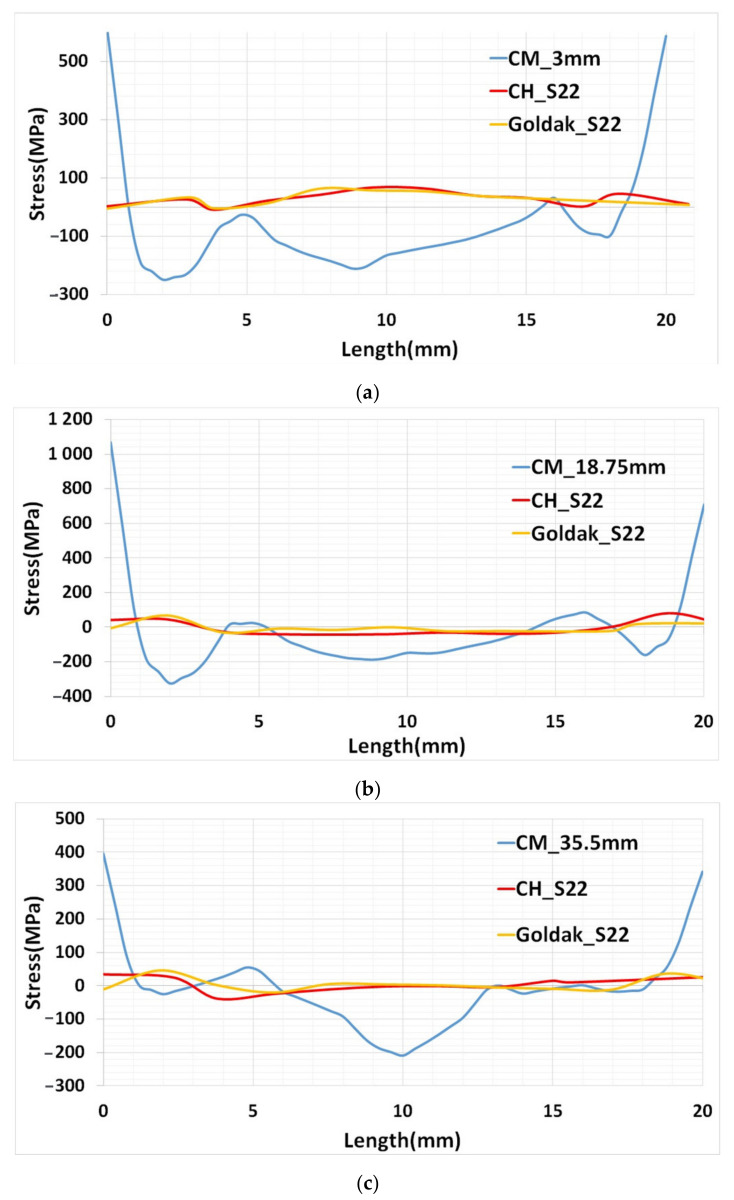
Residual stress comparison between simulation and experimental results (**a**) Path-1 (**b**) Path-2 (**c**) Path-3.

The field output for Y-component stress at three paths from the two-heat source model stress prediction was validated with the experimentally determined results from contour method. The results are depicted in Figure 17. The blue curve in the plot represents result from contour method and marked as “CM”. The numerical results from the concentrated heat source and Goldak model are represented as “CH” and “Goldak”, respectively.

The numerical results in Path-1 have a slight difference from the experimental results (Figure 17a). This could be due to the influence of the structural boundary condition during mechanical simulation. The structure was restricted to all three dimensions at the bottom of the base plate beneath the deposition area. The stress concentration in the base plate below deposition can be seen in Figure 16a,b. Path-2 has a good agreement with the experimental results (Figure 17b), whereas the Path-3 results show a difference in the middle section (Figure 17c). This difference could be reduced with a fine mesh model, where computation cost is a great concern with the fine-meshed model.

In all three cases, the stress calculation at the edge from the contour method has a large value as explained for the contour plot (Figure 16c). All three cases of residual stresses calculated from heat source model CH and Goldak has very close values. This shows the residual stress calculation from the CH model could be well-compared to the Goldak model.

#### 4.5.3. Temperature and Thermal Stress Evolution

The temperature evolution over different heights was analyzed to understand the influence on thermal stress during deposition process. The thermal simulation in the Section 4.5.1 revealed that temperature on the base plate gradually decreases as the deposition height increases, whereas the continuous interaction of laser with the depositing structure, results in a higher temperature in the building structure. Temperature in the different layers from the base plate, until the topmost layer was analyzed in this section. Five different layers were selected to compare the temperature evaluation during deposition process. Nodes in the 1st, 12th, 75th, 142nd and at 160th layers selected, and five nodes were marked with red dot in the Figure 16a.

**Figure 18:**
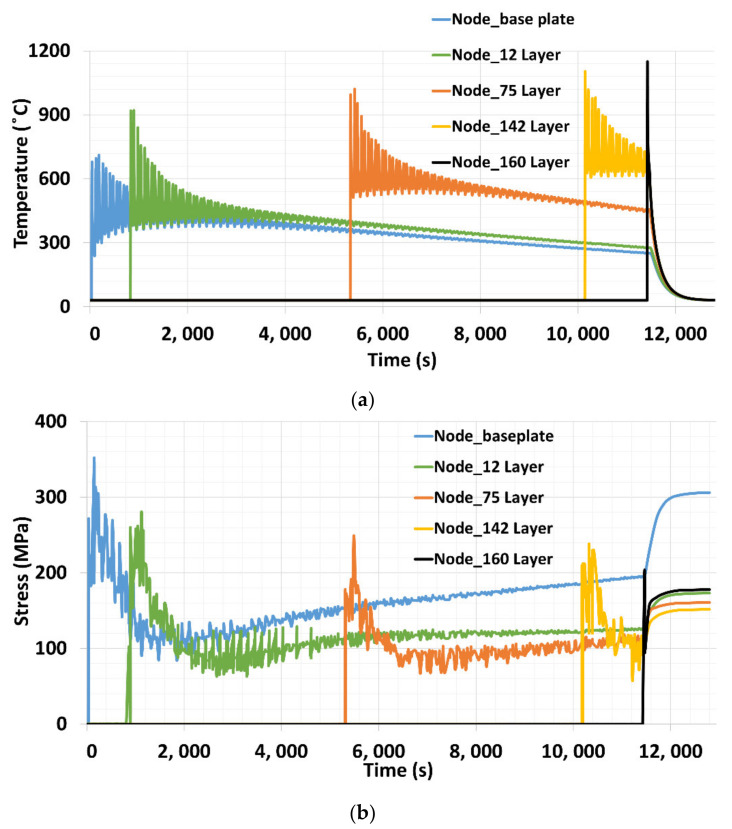
(**a**) Nodal temperature (**b**) Nodal stress at different layers.

Nodal temperature over the deposition time is depicted in Figure 18a. The maximum temperature developed during the first layer deposition was lower compared to the upper layers. Base plate at room temperature act as a strong sink that result in higher rate heat flow from the melt pool to the base plate, whereas the nodal peak temperature increases in the upper layers. Increasing nodal temperature over height could be due to continuous thermal source interaction with the deposition structure. Higher temperature in the layer act as preheated material for the next layer deposition. This could reduce the larger thermal gradient and, thus, thermal mismatch with underneath layer during material deposition.

The peak temperature in each node does not reach the melting point of the material steel 316 L (1400 °C), as shown in Figure 18a. The reason that the nodal peak temperature is less than the melting point of the material is due to course mesh and large increments. Course mesh was employed for reasonable computation time. A coarse mesh size reduces the number of elements and nodes, thus reducing the computational cost. Consequently, the element size is larger than the melt pool. Along with it, the activation time (increment) is often large, compared to a single-track to reduce the computation cost. This is one of the main reasons for nodal temperature output does not capture the melting point of the material, whereas the optimization was carried out to determine the ideal mesh size and increments. That results in both thermal and residual stress numerical output that were close comparison to the experimental data, as depicted in Figure 15 and Figure 17.

Similarly, the nodal stress at the corresponding layers were extracted from the mechanical simulation, as shown in Figure 18b. An inverse trend was observed for that of the nodal temperature. A maximum of 350 MPa was developed on the base plate at the intersection during first layer deposition. The peak stress of 280, 249, and 237 MPa was reported for nodes at the 12th, 75th, and 142nd layers, respectively. A node on the top surface records 202 MPa stress, which is the lowest, compared to the nodes in the previous layers. Interestingly, nodal stress on the base plate grows over the time and a drastic increase was reported during the cooling phase. The sharp rise in the stress at the end of deposition could be due to the interruption of the thermal source interaction with the structure. The highest thermal stress on the base plate during first layer deposition could be due to the large thermal gradient, developed when material deposits on the base plate at room temperature. Large temperature gradients, with the base plate at room temperature and elastic compression, imposed by the material around the melt pool results in the development of large thermal stress. Supporting these results, Park et al, reported a decrease in a thermal gradient from the bottom to the top layers at three positions [47]. Higher temperature at the upper layers results in a lower degree of restriction by surrounding material (beneath layer). This might be the reason for the lower-level thermal stress and reduction in the nodal peak stress, as depicted in Figure 17b.

## 5. Conclusions

A close comparison of thermal and mechanical simulation results for the concentrated heat and Goldak model was observed for single and multi-track deposition. The promising results for the CH model imply a strong stand in the AM thermal simulation.

The CH model shows a promising future for the following several reasons:

(1) The CH model is simple, as it does not require melt pool measurement. This directly reduces the complexity of numerical model preparation.
(2) A close agreement was reported for single-track CH model melt pool calculation. This model predicts the melt pool cross-section, with the precision of 87%, to the experimentally determined result, and melt pool depth measurement also falls within the measured data.
(3) This model saves enormous time for preprocessing. Noticeably, the computational time required for the CH model was less than half of that required for the Goldak model.
(4) This model is suitable for AM thermal simulation, as AM components contain several thousand overlapped weld tracks. It is economical, in terms of the time required for numerical model preparation, as well as the computational costs.
(5) The CH model results were a close match with the Goldak model for the 3D solid structure thermal and stress results. Therefore, the CH model would be an alternative for the Goldak model during thermo-mechanical AM process simulation.

Contradictory to the above benefits, the CH model has limitations:

(1) This model does not provide freedom for modification of melt pool definition. Though, in the present work, CH predicts melt pool in close agreement with the experimental results, this model might not be successful for process-level simulation. It is not recommended to apply for micro or mesoscale.
(2) This model is effective when the size of the finite elements used in thermal analysis is significantly larger than the size of the laser spot.

Further thermal stress evolution during the 3D solid structure deposition process over different heights was analyzed. Nodal thermal history reveals lower peak temperature in the layers near the base plate. Consequently, the thermal stress over deposition time indicates larger peak stress in the node near the base plate. These results specify the importance of base plate preheating. Base plate preheating could reduce the temperature gradient in the initial layer’s deposition. Base plate preheating would be the solution to reduce thermal stress in the initial layer deposition, as well as the overall residual stress in the deposited structure. These results encourage us to continue research on evaluating base preheating effects and numerical simulations, using a concentric heat source as a second part of this article.

## Figures and Tables

**Table 1:** Process parameter for DED.

Process Parameter	Cr
Laser power	500 W
Scanning speed	14 mm/s
Laser beam diameter	0.8 mm
Powder feed rate	3 g/min
Shielding and carrier gas	Argon
Shielding gas consumption	5 L/min
Laser standoff distance	9 mm

**Table 2:** Powder and base plate Steel 316 L chemical composition (wt.%).

	Fe	Cr	Ni	Mo	Mn	Si
Powder (316 L)	Bal.	17.2	10.4	2.3	1.3	0.8
Base Plate (316 L)	Bal.	16.24	10.49	2.14	1.12	0.44

## Data Availability

Data available in a publicly accessible repository.
